# Does online clinical mentoring for physical therapists enhance clinical practice and patient outcomes? A randomized controlled trial

**DOI:** 10.1080/10669817.2025.2481605

**Published:** 2025-03-27

**Authors:** Edmund Leahy, Lucy Chipchase, Rocco Cavaleri, Felicity C. Blackstock

**Affiliations:** a Physiotherapy, School of Health Sciences, Western Sydney University, Sydney, New South Wales, Australia; b Discipline of Physiotherapy, Department of Rehabilitation, Nutrition and Sport, La Trobe University, Melbourne, Victoria, Australia; c Physiotherapy Department, Northern Health, Melbourne, Victoria, Australia; d College of Nursing and Health Sciences, Flinders University, Adelaide, Australia; e Brain Stimulation and Rehabilitation (BrainStAR) Lab, School of Health Sciences, Western Sydney University, Sydney, New South Wales, Australia; f Office of the Deputy Vice Chancellor (Education), The University of Sydney, Sydney, New South Wales, Australia

**Keywords:** Mentoring, physical therapy, post-professional education, professional delvelopment, back pain, neck pain

## Abstract

**Objectives:**

The aim of this study was to determine whether a short-term online clinical mentoring program was more effective than asynchronous online lectures at improving physical therapists’ (PT) practice and their patients’ outcomes.

**Methods:**

In this randomized controlled trial, 27 PTs were randomized with allocation concealment to 6 h of online clinical mentoring sessions (experimental group) or 6-h of asynchronous online lectures (control group). The primary patient outcome was function, assessed using the Patient-Specific Functional Scale (PSFS), evaluated at baseline (initial consultation) and 4-week follow-up. Secondary patient outcomes were the Functional Rating Index (FRI) and Global Rating of Change Scale (GRC). Clinician (PT) outcomes were the ‘Clinician Confidence Questionnaire for Patients with Spinal Pain’ and the ‘Self-Reflection Insight Scale’, which were evaluated before and after the professional development interventions by blinded assessors. Linear mixed model regression analysis was used to explore differences in patient outcomes. PT outcomes were analyzed using analyses of covariance to control for any baseline differences.

**Results:**

Twenty-three PTs and 122 patients completed follow-up assessments. There were no between-group differences for any patient clinical outcomes (PSFS MD = 0.02, 95% CI −0.83, 0.79, *p* = 0.95; FRI MD = −3.01, 95% CI −10.71, 4.69, *p* = 0.42; Global Rating of Change MD = −0.08, 95% CI −1.09, 0.92, *p* = 0.86). There were also no differences between groups in terms of PTs confidence (MD = −2.17, 95% CI −9.11, 4.76, *p* = 0.52) or self-reflection insight (MD = 3.66, 95% CI −1.94, 9.27, *p* = 0.19).

**Conclusion:**

A 6-h online clinical mentoring program did not significantly influence PT confidence, self-reflection nor the outcomes of their patients when compared to 6 h of asynchronous online lectures.

**Impact:**

The results from this study may inform those designing or seeking professional development. Future online clinical mentoring should consider alternative program designs, target PTs with capacity to improve their patient outcomes, and evaluate effects on patients with chronic pain.

**Trial registration:**

ACTRN12622000123741

## Introduction

The development of clinical expertise throughout the career of a physical therapist (PT) requires commitment to continuing professional development (CPD) [1]. Accordingly, PTs are mandated to complete CPD hours annually to comply with registration and licensure requirements [2–5]. A recent systematic review indicated that clinical practice is enhanced when PTs engage in CPD with active learning components such as patient interactive sessions, peer review, mentored patient interactions, and feedback on observed performance [6]. In contrast, CPD events without active components, such as didactic lectures, have been shown to not change health professionals’ practice [7]. Active components are also perceived by PTs to be required for worthwhile learning, so need to be considered in CPD program design where the aim is to enhance PTs practice [8].

A CPD option that involves active learning components is clinical mentoring. During clinical mentoring, a clinical mentor provides feedback and guidance related to clinical practice [9]. Many evidence-informed learning strategies can be incorporated into clinical mentoring, such as self-evaluation, goal discussions, clinical reasoning communication, performance feedback conversations, and social learning facilitation [9–13]. Clinical mentoring has been used in post-professional education programs internationally for decades to assist PTs development of clinical expertise [14]. To truly understand if a CPD activity such as clinical mentoring is clinically impactful, rigorous research methods should be used to evaluate whether participating PTs’ patients’ outcomes improve [15,16].

Two previous randomized controlled trials (RCTs) report that participation in face-to-face clinical mentoring improves patient outcomes [17,18]. In the first study, 150 h of clinical mentoring was used [18], whereas the second study investigated the addition of 1 h of clinical mentoring and 3 h of group discussions to a two-day CPD course [17]. As patient outcomes in both studies improved despite considerable differences in time allocated for clinical mentoring, the optimum duration for effective clinical mentoring remains unclear.

While face-to-face clinical mentoring has evidence of clinical effect, it does not address the geographical and time barriers to participation often reported by PTs [8,19]. One viable solution is online clinical mentoring [9,20]. The one published study examining online clinical mentoring’s impact on PTs, reported that four-sessions improved confidence with clinical decisions and was perceived to improve CPD accessibility [9]. While these results are promising, patient outcomes were not evaluated, and there was no comparison group [9]. Thus, the impact of online clinical mentoring is yet to be completely determined. Whether the demonstrated clinical impact of face-to-face clinical mentoring translates to an online version of this CPD activity needs to be tested using rigorous methods and outcome measures.

### Aims

The primary objective of this study was to evaluate whether a six-session online clinical mentoring program was more effective than control (six 1-h prerecorded, structured sequence of online lectures) at improving PT confidence, self-reflection, and the clinical outcomes of their patients.

## Methods

### Study design and setting

This RCT was registered prospectively on the Australian New Zealand Clinical Trials Registry (ACTRN12622000123741) and the protocol published prior to recruitment [21]. Ethics was approved by Human Research Ethics Committees at Western Sydney University and La Trobe University (Approval number: H14629). Written informed consent was obtained from all participants.

The setting was 25 privately run PT practices located in Australia. PTs were randomized to an experimental (online clinical mentoring) or a control (online lectures) CPD group. To evaluate whether PT groups were comparable at baseline, the outcomes of patients with back or neck pain receiving care from each of the PTs were collected (cohort 1). Following the CPD intervention, patient outcomes were evaluated by collecting data from a different cohort of patients (cohort 2) receiving care from the PTs.

### Participant eligibility

#### Physical therapists

To be eligible for this study, PTs needed to have full Australian Health Practitioner Regulation Agency registration (license to practice), have less than 6 years experience, and be managing a patient caseload of at least three new patients with back or neck pain per week [21]. Participants needed to be planning to continue practicing as a PT with this caseload for at least 6 months. Physical therapists were excluded if they had completed, or were currently enrolled in, a post-professional, post-graduate musculoskeletal, or sports PT degree program that included mentored clinical practice.

#### Patients

Patients of participating PTs were recruited to evaluate clinical outcomes. To be eligible, patients needed to have back or neck pain (± limb pain) of any duration. Patients also needed to be seeing the PT for the first time due to the current back or neck pain episode. Patients were excluded if they had a suspected or confirmed diagnosis that would contra-indicate PT management and require medical referral. Such diagnoses included, but were not limited to, vertebral fracture, abdominal aortic aneurysm, or cancer. Patients were excluded when English language proficiency or cognitive function limited their ability to consent and/or complete questionnaires.

### Interventions

Experimental and control groups completed 6 h of online CPD over 6 weeks. This duration of CPD was chosen because previous research reported that an online clinical mentoring program of a similar duration may positively impact PT clinical practice [9]. A schedule for the CPD groups has been published previously [21].

#### Online clinical mentoring (experimental CPD intervention)

The experimental group completed six online clinical mentoring sessions. Each session was 1 h in duration. Clinical mentoring was provided by Specialist Musculoskeletal PTs as awarded by the Australian Physiotherapy Association. The mentors had on average (SD) 31 (13.94) years of clinical experience and 22.4 (12.58) years of experience teaching and providing clinical mentoring in structured post-professional musculoskeletal PT programs accredited by the International Federation of Orthopedic Manipulative Physical Therapists.

Online clinical mentoring consisted of two consecutive phases. Phase one consisted of four group sessions using a three-learners to one clinical mentor ratio. Phase two consisted of two sessions with the same clinical mentor, using a one learner to one mentor ratio.

For phase one, the first three online clinical mentoring sessions were based on case studies presented by each PT learner. During the sessions, PTs were encouraged to share thoughts, perspectives, and feedback regarding the case to the presenting PT. During the final group clinical mentoring session, each PT provided a brief reflection on how they incorporated learnings into clinical practice, and barriers encountered. Clinical mentors facilitated discussion, shared perspectives, corrected misconceptions, and guided PTs toward resources to further extend their knowledge.

For phase two, each PT completed a video-recorded interaction with a patient with spinal pain. The interaction was recorded on the PT’s personal device and uploaded onto a password protected cloud folder. The PT also completed a clinical reflection form adapted from a previous publication [22]. The clinical mentor reviewed the recording and reflection form before the clinical mentoring sessions. During the initial one-to-one session the PT discussed their self-evaluation, requested performance feedback, discussed feedback provided, formulated learning goals, and discussed strategies to assist goal achievement. Discussions and feedback continued during the second session, where PTs reflected on their progress toward goal achievement. Collaborative problem solving of implementation barriers were explored with the clinical mentor.

Strategies were implemented to ensure that clinical mentoring was delivered as intended. These strategies included providing education to the clinical mentors and have been published previously [21].

#### Online lectures (control)

The control group watched six prerecorded online lectures (1 h each, one per week) and completed 4 h of self-directed reading of peer reviewed publications relating to clinical guidelines and spinal pain care [23–26]. Content of the first three lectures related to the evidence-based principles of asking questions, searching for evidence, critical appraisal, and interpreting results [27]. The final three lectures covered recently published clinical practice guidelines for differential diagnosis and management of neck and back pain disorders [23–26]. There were four compulsory readings [23–26], and participants could choose from 18 additional readings to read, making up the total of 4 h required. Online lectures were chosen for the control group as high-level evidence indicates that didactic lectures are ineffective at influencing health care professionals’ behavior [6,7].

Lectures were prerecorded by an educator with 25 years of clinical experience and 15 years of experience teaching evidence-based practice and spinal pain physical therapy in clinical and academic settings. A participant guide was provided (details available in the published protocol) [21].

Control group participants were instructed to watch one online lecture per week for 6 weeks and complete self-directed tasks at times convenient to them. The participants completed a self-evaluation checklist of the required tasks which was used to assess instruction adherence.

### Adherence monitoring procedures

The degree to which clinical mentoring was delivered as intended was evaluated using a ‘clinical mentoring fidelity checklist’ based on a published set of 25 educator behaviors recommended for high-quality feedback [28] and described in the study protocol [21]. Each clinical mentoring session was video-recorded, with two sessions per clinical mentor evaluated by the lead researcher (EL).

### Data collection and outcomes

**Figure 1.**
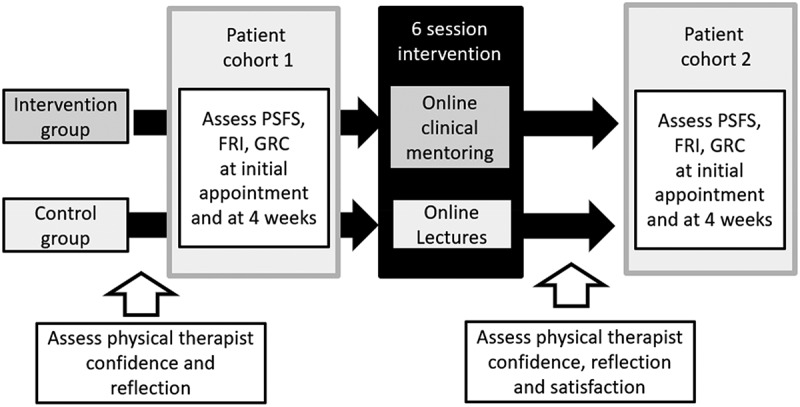
Research design and outcome measure timing.

Data collection and outcome measurement was completed at the patient and PT levels. An explanation and justification for patient and PT outcome measures is in the published study protocol [21]. A timeline of outcome measure collection is illustrated in Figure 1.

PSFS = Patient-Specific Functional Scale, FRI = Functional Rating Index, GRC = Global Rating of Change

#### Patient outcomes

The primary outcome for the relative effectiveness of the CPD activities was the functional change of patients under the PTs’ care post-CPD intervention completion (cohort 2). This was evaluated using the Patient-Specific Functional Scale (PSFS), which has concurrent validity in relation to the Roland Morris Questionnaire (*r* = 0.55 to 0.74), Neck Disability Index (*r* = 0.82), Numeric Pain Rating Scale (*r* = 0.80), and Global Rating of Change scale (GRC) (*r* = 0.82) [29–32]. The PSFS has fair to moderate (intra- or inter-rater) reliability when used for back or neck pain (ICC = 0.52 to 0.91) [29–31]. Secondary patient outcomes were the Functional Rating Index (FRI) and Global Rating of Change (GRC). The FRI has limited positive evidence of validity and moderate to excellent reliability (ICC = 0.63 to 0.948) [33]. The GRC has high face validity and excellent reliability (ICC = 0.90) when used with patients experiencing neck or back pain [34].

Patient contact, demographic and baseline data were collected by participating PTs at initial appointment. A research team member then contacted the patients by phone 4 weeks after the initial appointment to complete patient outcomes. Four weeks were sufficient time to see between-group differences in patient outcomes in a previous PT CPD study [35]. Patients not available for a phone call, were offered an online survey to provide follow-up data.

#### Physical therapist outcomes

Secondary outcomes evaluated PT clinical confidence and self-reflection. Clinical confidence was evaluated using the ‘Clinician Confidence Questionnaire for Patients with Spine Pain’ [14]. Self-reflection was evaluated using the Self-Reflection Insight Scale (SRIS) [36,37]. In addition to the secondary outcomes, satisfaction with the CPD was assessed using a 5-point Likert scale.

### Sample size

Sample size calculations were based on the planned linear mixed model analysis using GLIMMPSE software (https://glimmpse.samplesizeshop.org/). The primary outcome of PSFS expected between-group mean difference at 4-week post-patient intervention was set at 1.0 with a standard deviation of 2.0 [18,29,30,32,38]. Statistical significance was set at 0.05, power at 0.80 and ICC at 0.05. The estimated sample size for achieving this with the primary outcome of PSFS was 182 patients. To account for dropouts of 14%, and achieve power, the final sample size aimed for was 210 patients. Based on previous research, it was estimated that each PT would recruit approximately seven patients. Thus, the target sample consisted of 30 PTs and 210 patients. These sample size calculations only applied to patients seen after the CPD intervention (cohort 2).

### Recruitment, randomization, and blinding

Snowball recruitment of PTs was completed through e-mails to private practices, and social media postings. Physical therapists expressing interest and fulfilling the inclusion criteria were invited to participate. Each participating PT was asked to recruit consecutive consenting patients (cohort 1) before completing the CPD intervention. Following the completion of the CPD, they were asked to recruit another nine consecutive consenting patients (cohort 2).

The PTs were randomized in permuted blocks of six using a random number generator. Group allocation was concealed by having an offsite researcher (FB) not involved in recruitment or data collection informing participants of their group allocation. Which CPD was planned for each group was not divulged to the allocating researcher.

Participants were blinded to whether they were in the intervention or control group and were asked not to discuss the study until outcome measures were collected. Blinding success was evaluated by asking PTs whether they thought they were in the intervention or control group after collection of all outcome measures. All patient outcomes were collected by researchers blinded to group allocation (RC and those acknowledged).

### Statistical methods

Data analyses were conducted using SPSS statistical software and used intention-to-treat principles with alpha set at 0.05. Analysis included the calculation of between-group point estimates and 95% confidence intervals [39].

Patient outcomes of PSFS, FRI, and GRC were analyzed using a linear mixed model, which inherently accounts for missing values [40]. The treatment effects were estimated from the post-treatment group means (intervention versus control), with adjustment for outcome measure baseline score. The analysis was performed with adjustment for baseline outcome score (minimally adjusted) and with adjustment for age, gender, pain duration, pain area, funding (compensable or not compensable), and Orebro score (maximum adjustment). Where possible, the PTs were included in the model as a random effect and the patients clustered within each PT. Where convergence was not possible due to PTs not accounting for variation, the analysis was completed as a univariate linear model analysis. Additional linear mixed model analyses were completed to determine if there were between-group differences in the change in patients’ outcomes for the PSFS before (cohort 1) and after (cohort 2) completion of the CPD.

To enable comparisons with previous studies [17,18,35], the proportion of patients from each group achieving the minimally clinically important difference (MCID) was determined. Patient reported outcomes were dichotomized as to whether they achieved the MCID which was defined as 2 for the PSFS, 8.4 for FRI and 2 for GRC [29,30,32,33,41]. Risk ratio, risk difference and numbers-needed-to-treat were calculated using an online calculator (https://ebm-tools.knowledgetranslation.net/calculator/prospective/). Consistent with previous work, logistic mixed model analysis was planned to be used to determine statistical significance of the dichotomized PSFS, FRI, and GRC [18,42]. Convergence of the model was not possible for any dichotomized outcome due to a lack of variation attributable due to PTs. Instead, a logistic regression analysis was completed, without the inclusion of the PTs as a random factor. The PT outcomes of confidence, self-reflection, and satisfaction were analyzed using an ANCOVA which adjusts for any baseline differences and is a complete-case analysis.

## Results

**Figure 2.**
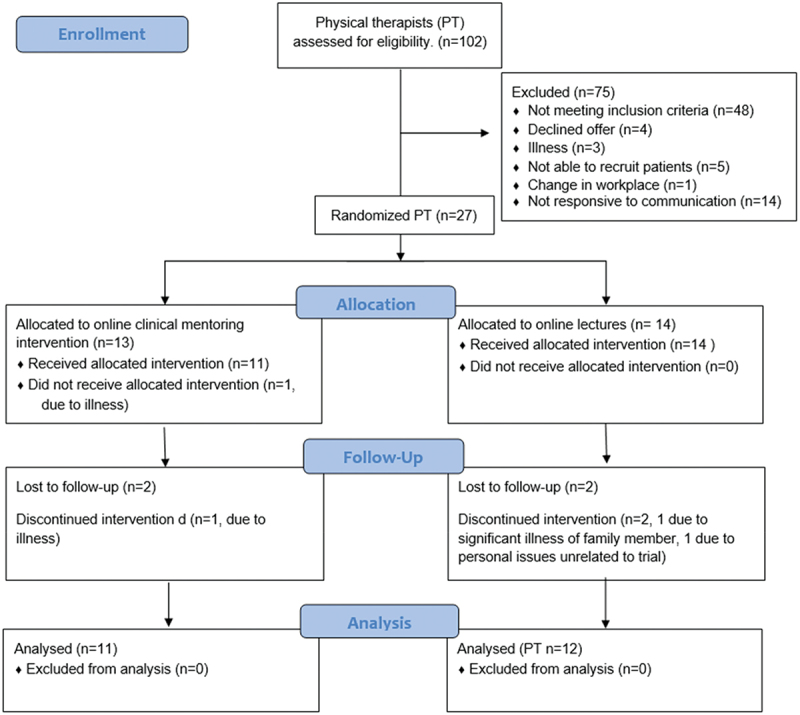
Consort diagram.

Participant recruitment commenced on 6 November 2022 and concluded on 15 November 2023. Of the 102 PTs expressing interest in the study, 27 were randomized to one of the professional development activities. Reasons for exclusion are presented in Figure 2. The 27 participating PTs were 17 males and 10 females. There were no differences between groups for workplace or age (Table 1). There was a statistically significant difference between groups for years of experience, however this was only 1.04 years (mean (SD) intervention = 2.46 (1.26), control = 1.42 (1.29)). Mean time taken by PTs to complete all activities required by the study was 34.91 weeks (SD 10.46, range 17.86, 55.14). Two PTs from each group were lost to follow-up (14.8%) due to reasons unrelated to the study.

**Table 1.** Physiotherapist participants demographics and patient PSFS change scores before the educational interventions.

	Intervention group	Control group	*p* value
Gender	6 M; 7 F	11 M; 3 F	p = 0.12^a^
Workplace	11 Metropolitan, 2 regional	10 Metropolitan, 4 regional	*p* = 0.65^a^
	**Mean (SD)**	**Mean (SD)**	
Age (years)	26.23 (1.83)	26.14 (2.63)	*p* = 0.92^b^
Experience (years)	2.46 (1.26)	1.42 (1.29)	*p* = 0.046 ^b, c^
Mean (SD) scores for change in PSFS from initial appointment to 4-week follow-up pre-educational intervention.	2.89 (2.44)	3.55 (2.69)	*p* = 0.12 ^b^

M = Male; F = Female; SD = Standard deviation; PSFS = Patient Specific Functional Scale.

^a^Fishers Exact test.

^b^Independent samples t test.

^c^Statistically significant.

One-hundred and sixty-five patients (6.11 per PT) were recruited by the PTs prior to the CPD (cohort 1) with 149 completing follow-up assessments. One hundred and fifty patients (5.56 per PT) were recruited by the PTs after completion of the CPD (cohort 2) with 119 completing follow-up assessments. There were no differences at baseline between patient groups (Table 2) on visual inspection analysis of means and proportions. Independent t-tests and χ^2^ tests confirmed similarities between groups, except for cohort 1 (pre-CPD) age and gender. These differences were not considered clinically important, so no changes in analysis procedures were implemented. In addition, there were no differences between the groups at baseline for the primary outcome of change in PSFS of patients seen by the PTs (Table 1).

**Table 2.** Patient characteristics.

		Pre CPD (cohort 1)			Post CPD (cohort 2)		
Variable		Intervention	Control	p	Intervention	Control	p
		N (%)	N (%)		N (%)	N (%)	
Gender	Male	32 (39.5)	48 (57.1)		25 (33.8)	34 (44.7)	
	Female	49 (60.5)	36 (42.9)	0.02^a^	49 (66.2)	42 (55.3)	0.17
	*Missing*						
Area of pain	Back	39 (48.1)	52 (61.9)		38 (51.4)	48 (63.2%)	
	Neck	13 (16)	16 (19.0)		15 (20.3)	13(17.1)	
	Neck back	4 (8.6)	5 (6.0)		9 (12.2)	6 (7.9)	
	Spine peripheral	11 (12.9)	22 (27.5)	0.68^b^	9 (11.8)	12 (16.2)	0.64^b^
	*Missing*						
Funding	Compensable	7 (8.2)	8 (10)		12 (15.8)	5 (6.8)	
	Not compensable	78 (91.8)	72 (90)	0.69	64 (84.2)	69 (93.2)	0.81
	*Missing*						
Duration of pain	Acute	40(49.4)	45 (53.6)	0.76	33 (44.6)	42 (55.3)	0.18
	Subacute	13 (16)	13 (15.5)		10 (13.5)	13 (17.1))	
	Chronic	28 (34.6)	26 (31.0)		31 (41.9)	21 (27.6)	
	*Missing*						
		**Mean (SD)**	**Mean (SD)**	**p**	**Mean (SD)**	**Mean (SD)**	p
Age		45.35 (17.54)	54.62 (16.41)	0.002^a,c^	43.97 (19.14)	47.62 (18.2)	0.23^d^
	*Missing*						
Orebro		46.45 (13.95)	46.66 (13.70)	0.93	47.49 (14.53)	47.18(14.53)	0.85
	*Missing*	1	1				
PSFS		4.24 (1.95)	4.33(1.93)	0.67^c^	4.18 (1.78)	4.38(1.97)	0.61
	*Missing*				1	1	
FRI		47.64 (18.02)	50.63 (18.22)	0.29	50.68(20.01)	49.91 (19.70)	0.79
	*Missing*						

CPD = Continuing professional development; PSFS = Patient Specific Functional Scale; FRI = Functional Rating Index; p = p-value; SD = Standard deviation.

^a^Statistically significant.

^b^To calculate the X^2^ the neck back group and spine peripheral categories were combined to a multi-area category to ensure cells had > 5 subjects each.

^c^Non-normal distribution on Shapiro-Wilk test, approximately normal on visual inspection of histogram.

^d^Non-normal distribution on Shapiro-Wilk test and on visual inspection.

### Patient outcomes

In the minimally adjusted linear model analysis, there were no differences between the groups for the patient PSFS at 4-week follow-up in cohort 2 (MD = 0.02, 95% CI −0.83, 0.79, *p* = 0.52). The clinical mentoring group had within group improvements in PSFS from cohort 1 to cohort 2, however these did not reach the MCID of 2. (See Supplementary material: Table 5 and Figure 3.) There were also no between-groups differences for the FRI or GRC outcomes at 4-week follow-up (MD = −3.01, 95% CI −10.71, 4.69, *p* = 0.42; MD = −0.08, 95% CI −1.09 0.92, *p* = 0.86). No between-group differences were found on completing the analysis with the fully adjusted model (Table 3.)

**Table 3.** Patient outcomes and statistical analysis results for cohort 2.

	Clinical mentoring	Lectures	Linear regression model					
Patients	56	63	Minimally adjusted^b^			Fully adjusted^c^		
	Outcome mean at 4 weeks (SD)^a^		MD	95% CI	p	MD	95% CI	p
PSFS	7.51 (2.11)	7.56 (1.87)	0.02	−0.83, 0.79	0.52	0.20^*e*^	−0.49, 0.89	0.49^*e*^
FRI	24.87 (19.66)	28.45 (20.52)	−3.01	−10.71, 4.69	0.42	−4.71	−12.12, 2.7	0.19
GRC^d^	2.89 (1.98)	2.89 (1.71)	−0.08	−1.09, 0.92	0.86	0.09	−0.94, 1.13	0.85

PSFS = Patient Specific Functional Scale, FRI = Functional Rating Index, GRC = Global Rating of Change, MD = mean difference, CI = confidence interval, p = p-value.

^a^Means calculated from raw scores and unadjusted.

^b^Adjusted for baseline differences (pre-physical therapy intervention outcome score).

^c^Adjusted for sex, age, symptoms duration, Orebro, area of symptoms, compensation.

^d^No baseline score to adjust for.

^e^Physical therapist unable to be included as random factor in this analysis.

For the change in the PSFS primary outcome from cohort 1 to cohort 2, the linear mixed model analysis found no between-group differences (minimally adjusted analysis *p* = 0.20, MD = 0.79, 9% CI −0.43, 2.02; fully adjusted analysis, *p* = 0.07, MD 1.06, 95% CI −0.07, 2.19). (Supplementary material.)

Logistic analysis of dichotomized patient outcomes for cohort 2 with respect to reaching the MCID, also found no differences between groups. This was confirmed by inspection of the calculated relative risk, absolute risk reduction, and numbers-needed-to-treat. (Supplementary material.)

### Physical therapist outcomes

Both groups improved by more than 14% (19 points) on the Clinical Confidence Questionnaire for Patients with Spinal Pain, however there were no differences between groups (F = 0.43, df 1, error 20, 95% CI −9.11 to 4.76, *p* = 0.52). There were also no differences between groups for the SRIS (F = 1.88, df 1, error 19, 95% CI −1.94, 9.27, *p* = 0.19) or PT satisfaction with learning (F = 4.2, df 1 error 21, 95% CI −0.002, 0.94, *p* = 0.051) (Table 4.)

**Table 4.** Mean scores and ANCOVA results for clinician outcomes before and after professional development interventions.

	Pre-CPD		Post-CPD			
Measure	Clinical mentoring	Lectures	Clinical mentoring	Lectures	MD (95% CI)	p
Clinician Confidence Questionnaire for Patients with Spinal Pain	83.23 (11.54)	83.14 (12.54)	104.73 (7.77)	106.14 (8.31)	−2.17 (−9.11, 4.76)^a^	0.52
Self-Reflection Insight Scale	81.77 (10.30)	78.78 (8.81)	85.45 (8.01)	79.00 (13.33)	3.66 (−1.94, 9.27)^a^	0.19
Satisfaction with learning activity	Notcollected	Not collected	4.64 (0.51)	4.15 (0.58)	0.47 (−0.002, 0.94)	0.051

CPD = continuing professional development; MD = mean difference; *p* = p-value.

^a^Adjusted for baseline means.

### Fidelity of clinical mentoring

Using the clinical mentoring checklist, 10 online clinical mentoring sessions were evaluated, two for each of the five mentors. Of the 21 mentoring behavioral indicators on the checklist, 12 were observed in most sessions (scoring of ≥2/3 on the checklist in at least 7/10 sessions). These observed items are related to mentor feedback delivery, feedback prioritization, suggestions of alternative perspectives, mentees articulating thought processes, and discussions based on mentee questions. The nine items that were observed inconsistently related to explanations of the mentoring process, feedback dialog, learning goal discussions, learning implementation strategies and mentees verbally communicating self-evaluations.

## Discussion

This study was the first to evaluate the effectiveness of interactive online clinical mentoring sessions compared to a control (online prerecorded lectures). There was no observed additional benefit of online clinical mentoring over the online lectures in terms of PT patient outcomes. The six-session program of online clinical mentoring also had no observable effect on PT confidence or self-reflection insight when compared to online lectures. Overall, these findings suggest that the design of a brief online clinical mentoring program, as used in this study, is insufficient to influence PT practice for improved patient outcomes.

While findings from this study suggest that online clinical mentoring may not be the most effective CPD approach, this does not mean that PTs did not learn and develop awareness to improve practice. The Awareness-to-Adherence model considers learning as a process where a clinician works through four learning phases before achieving a sustained change in their practice [43,44]. In the initial awareness phase, the clinician becomes aware of different ways of practicing clinically. This is followed by the agreement phase, where the clinician decides that the learning should be incorporated into clinical practice. In the adoption phase, the clinician implements what was learned on an irregular basis. In the final adherence phase, the clinician consistently incorporates learnings into clinical practice [43,44]. It is possible that mentees had inadequate time to develop sustained adherence to practice change to the extent that improvements in patient outcomes could be measured. While not directly measured, this theory is supported by our checking of clinical mentoring fidelity that found inconsistent observations of adherence strategies, such as discussions of learning goals and implementation barriers. Future studies should consider examining outcomes related to the four stages of the Awareness-to-Adherence model, in conjunction with patient outcomes, to determine whether online clinical mentoring of short duration influences earlier stages of learning.

Awareness-to-adherence and long-term practice change may require more than 6 h of clinical mentoring. Indeed, results from the current study are different from previous clinical mentoring studies where PTs participated in face-to-face learning activities over longer periods. One-hundred and fifty h of face-to-face clinical mentoring was found to be effective in a study by Williams, Rushton, Lewis, Phillips [18]. Similarly, Cleland, Fritz, Brennan, Magel [17] found combining a two-day CPD course with two additional interactive sessions, and 1 h of face-to-face clinical mentoring improved patient outcomes. Longer time spent in supported learning activities in these studies may have enabled mentees to be supported through all four learning phases, including the final adherence phase. Additionally, in-person learning may require differing time durations to reach the adherence phase than online learning, hence the need to evaluate these delivery modes separately. Future research could explore whether online clinical mentoring models with learning activity durations longer than 6 h, spaced over time, are associated with consistent use of adherence strategies during mentoring sessions and improve patient outcomes.

Contextual factors, such as patient condition, may have influenced outcomes and should be considered with future clinical mentoring research. Half the patients in this study had acute pain, which differs to the effective study by Williams, Rushton, Lewis, Phillips [18] who recruited only patients with chronic conditions. Chronic conditions bring complexity requiring multidimensional management, which in turn requires a more sophisticated clinical reasoning approach for patient management [45–48]. Professional development for complex clinical reasoning is grounded in a multi-dimensional knowledge base, and higher-level cognitive capabilities [49,50] Clinical mentoring provides experiential learning approaches that enable development of this higher order thinking [50]. Future studies evaluating online clinical mentoring should consider focusing on patients with chronic conditions to demonstrate impact and avoid ceiling effects.

Care is needed when generalizing this study’s findings to the general PT population, due to the participating PTs’ high level of baseline patient outcomes (cohort 1). The PTs in this study achieved an average treatment effect higher than the MCID for the PSFS before completing clinical mentoring. (Supplementary Material.) As such, the participating PTs may have already been practicing in an effective manner, therefore the results may not be generalizable to PTs whose patient outcomes demonstrate improvement lower than the MCID. Prior to commencing clinical mentoring, no learning needs assessment was completed to identify whether CPD was required to improve outcomes of participating PTs’ patients with back or neck pain. Therefore, PTs in this study may already have a ceiling on their potential improvement, and further treatment effects may not have been possible. Future research should consider targeting CPD based on identified learning needs and opportunities to improve patient outcomes.

Finally, PTs in this study were motivated to participate in a research project over many months, so may not be representative of other PTs in clinical practice. This motivation was evidenced by PTs completing all required tasks over an average of 34.91 weeks. In addition, 60% of PTs completing the study reported they were engaged in regular clinical mentoring in their workplace. This engagement, prior to and during the study, may have influenced the outcomes. This highlights the difficulties of this type of research design. Learning by highly motivated clinicians may occur in ways that cannot be controlled. Hence, future research should consider reducing participation barriers for less motivated PTs, such as completing online clinical mentoring during paid work hours.

This study employed a rigorous approach toward blinding and statistical analyses; however, several limitations require consideration. The sample size was less than that calculated in the *a-priori* power analysis. Fortunately, this sample size was still sufficient to determine clinical impact, as the mean differences and confidence intervals for the primary outcome of PSFS (MD = 0.02, 95% CI −0.83, 0.79) did not reach the MCID of 2. This indicates no clinically impactful differences, with a high degree of certainty [51], and increasing the sample size for sufficient power at such a small effect size is unlikely to change this conclusion. The minimal training of the mentors is a possible limitation, as a more comprehensive mentoring training program may have led to greater fidelity and subsequently improved outcomes. This assumes improved mentoring fidelity is associated with improved outcomes, which has not yet been established. Hence, future research should examine whether this relationship exists before determining the best way to train the mentors. Additional study limitations included patient loss-to-follow-up, PT loss-to-follow-up and the potential for bias related to motivation or additional CPD completion beyond the study, as outlined in the previous paragraphs. This study highlights the inherent challenges to completing educational intervention research in the clinical practice context, where confounding variables are difficult to control and competing workload priorities for clinicians may impact upon a study’s rigor. In future research, steps should be taken to minimize barriers to PT participation. These may include strategies to reduce data collection burden such as online data collection, minimizing volunteer recruitment of patients and funded data collection processes.

## Conclusions

In a private practice setting, a 6-h online clinical mentoring program was insufficient to influence PT confidence and patient outcomes when compared to online lectures. Additional research is required to determine whether different designs of online clinical mentoring programs, such as combining online clinical mentoring with short courses focused on knowledge and skills, and programs of online clinical mentoring of longer durations, lead to greater use of adherence strategies and improved patient outcomes. Future research should also consider limiting outcome measurement to patients with chronic pain, evaluating additional outcomes that measure learning phases, and identifying PTs’ learning needs so that professional development can be targeted to those with the capacity to improve practice.

## Supplementary Material

Supplemental Material
