# Supplementary material for: Does online clinical mentoring for physical therapists enhance clinical practice and patient outcomes? A randomized controlled trial
Source: J Man Manip Ther. 2025 Mar 27;33(6):490–500. doi: 10.1080/10669817.2025.2481605 (PMC12624911; doi:10.1080/10669817.2025.2481605)

# Supplementary Material:

## Table 5. Patient specific functional scale scores before (cohort 1) and after (cohort 2) the professional development interventions.

|  | **Pre professional development intervention**  **(Cohort 1)** | | | | **Post professional development intervention**  **(Cohort 2)** | | | |
| --- | --- | --- | --- | --- | --- | --- | --- | --- |
|  | **Patients** | **PSFS score first visit** | **PSFS score follow up** | **Mean change PSFS scores** | **Patients** | **PSFS score first visit** | **PSFS score follow up** | **Mean change PSFS scores** |
|  | **N** | **Mean (SD)** | **Mean (SD)** | **Mean (SD)** | **N** | **Mean (SD)** | **Mean (SD)** | **Mean (SD)** |
| Clinical mentoring | 76 | 4.22 (1.95) | 7.167 (2.08) | 2.91 (2.45) | 56 | 4.18 (1.77) | 7.51 (2.11) | 3.42 (2.69) |
| Lectures | 73 | 4.35 (1.93) | 7.84 (1.79) | 3.52 (2.69) | 63 | 4.34 (1.97) | 7.56 (1.87) | 3.25 (2.20) |

PSFS = Patient specific functional scale

Means and SD calculated from raw scores and unadjusted.


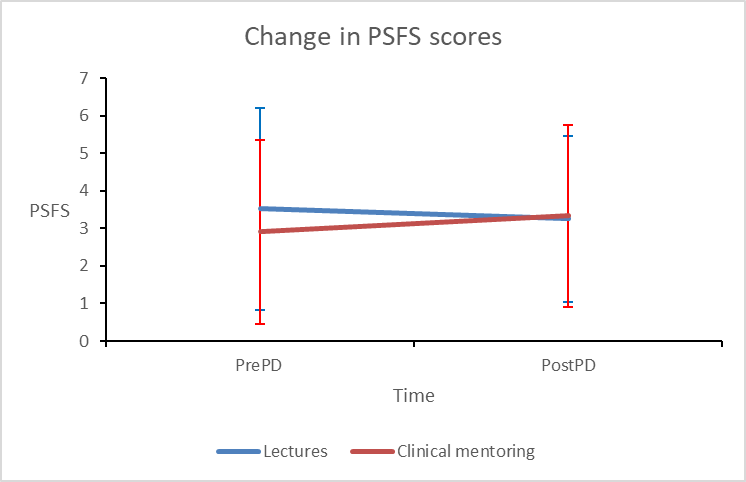


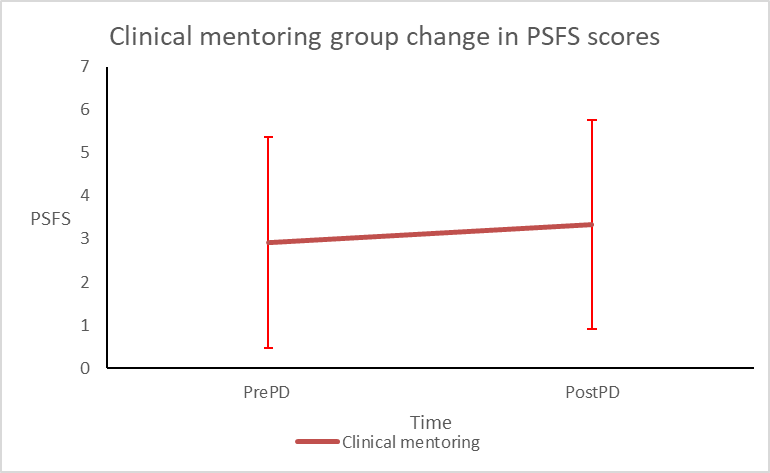


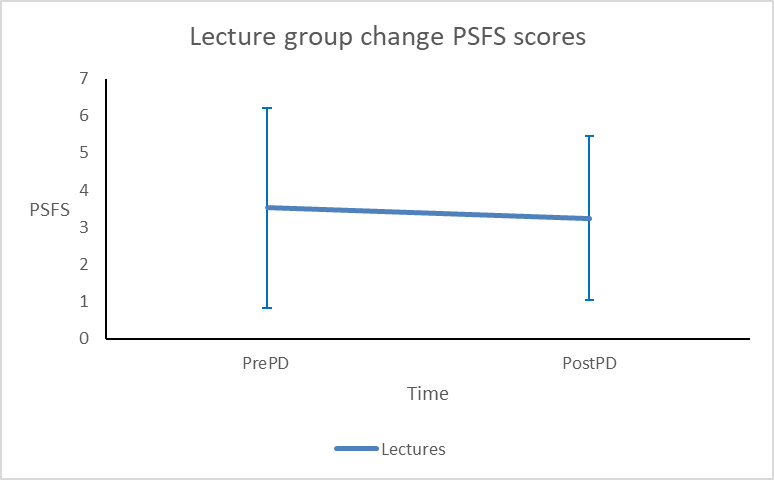


## **Figure 3.** Change in PSFS, pre-professional development (cohort 1) and post-professional development interventions (cohort).

## Table 6. Analysis of dichotomised patient outcomes for cohort 2

|  | **Clinical mentoring** | **Lectures** | **Logistic regression model**  **p-value** | | **Descriptive statistics**  **Mean difference (confidence interval)** | | |
| --- | --- | --- | --- | --- | --- | --- | --- |
| N | 56 | 63 | **Not adjusted** | **Fully adjusted^b^** | **Relative Risk** | **Absolute Risk Reduction** | **Numbers Needed to Treat** |
|  | MCID outcome at 4 weeks*^a^*  N (%) | |  |  |  |  |  |
| PSFS | 39 (70%) | 46 (73%) | 0.80 | 0.77 | 0.97 (0.77, 1.22) | 0.02 (-0.14, 0.18) | 47 (-7, 5) |
| FRI | 37 (66.1%) | 47 (74.6%) | 0.30 | 0.39 | 0.87 (0.69, 1.10) | 0.1 (-0.06, 0.26) | 10 (-15, 4) |
| GRC | 51 (81%) | 44 (78%) | 0.75 | 0.73 | 0.97 (0.81, 1.16) | 0.02 (-0.12, 0.17) | 42 (-8, 6) |

*PSFS = Patient Specific Functional Scale, FRI= Functional Rating Index, GRC= Global Rating of Change, MCID = Minimally clinically important difference.*

*^a^Calculated from raw scores and unadjusted.*

*^b^Adjusted for sex, age, symptoms duration, Orebro, area of symptoms, compensation.*

**APPENDIX**


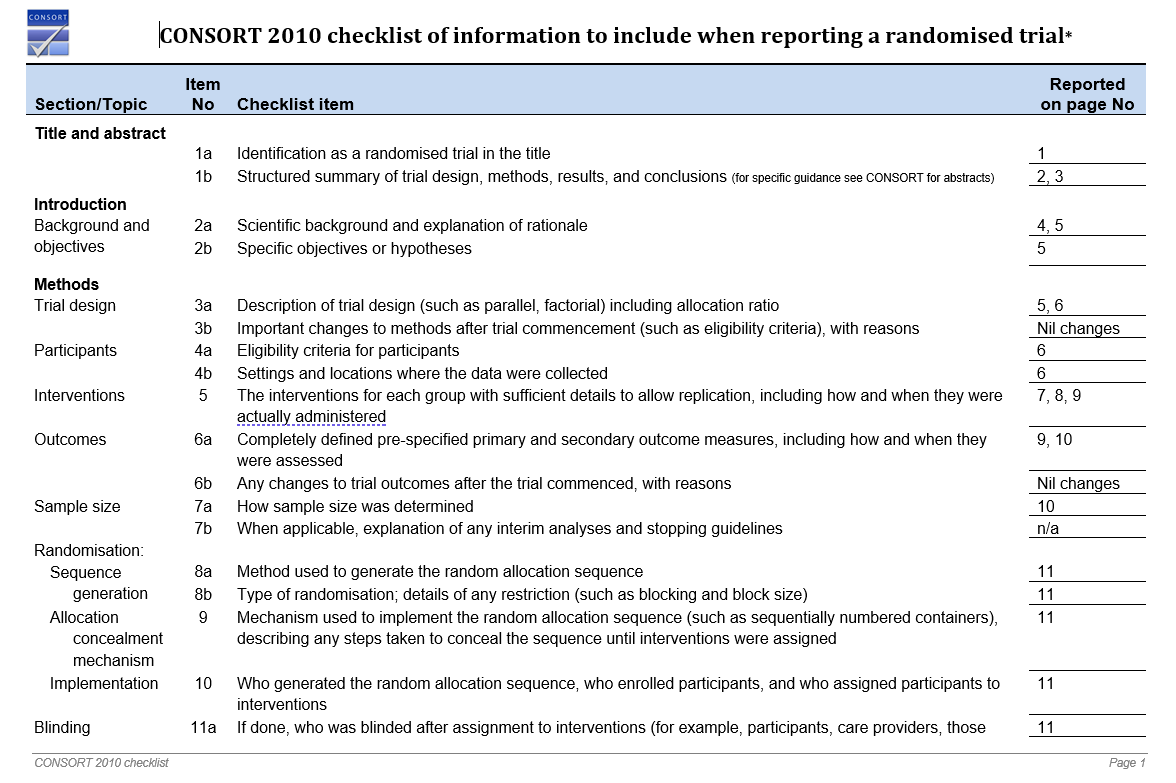


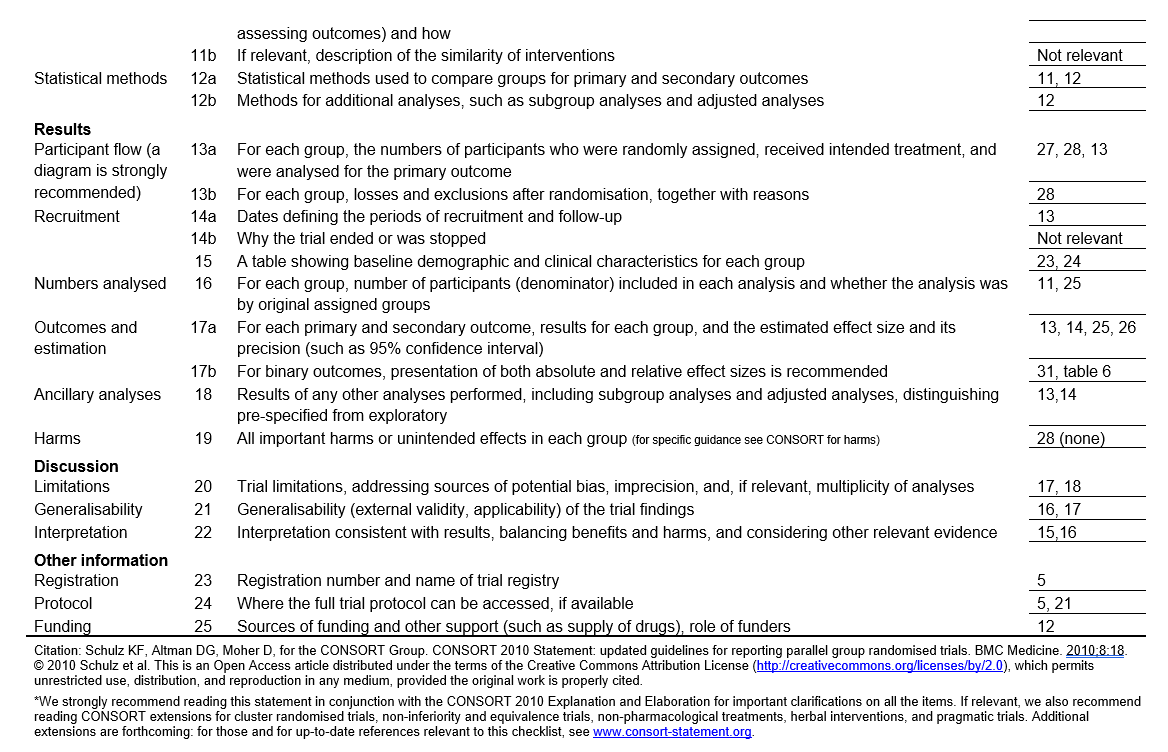


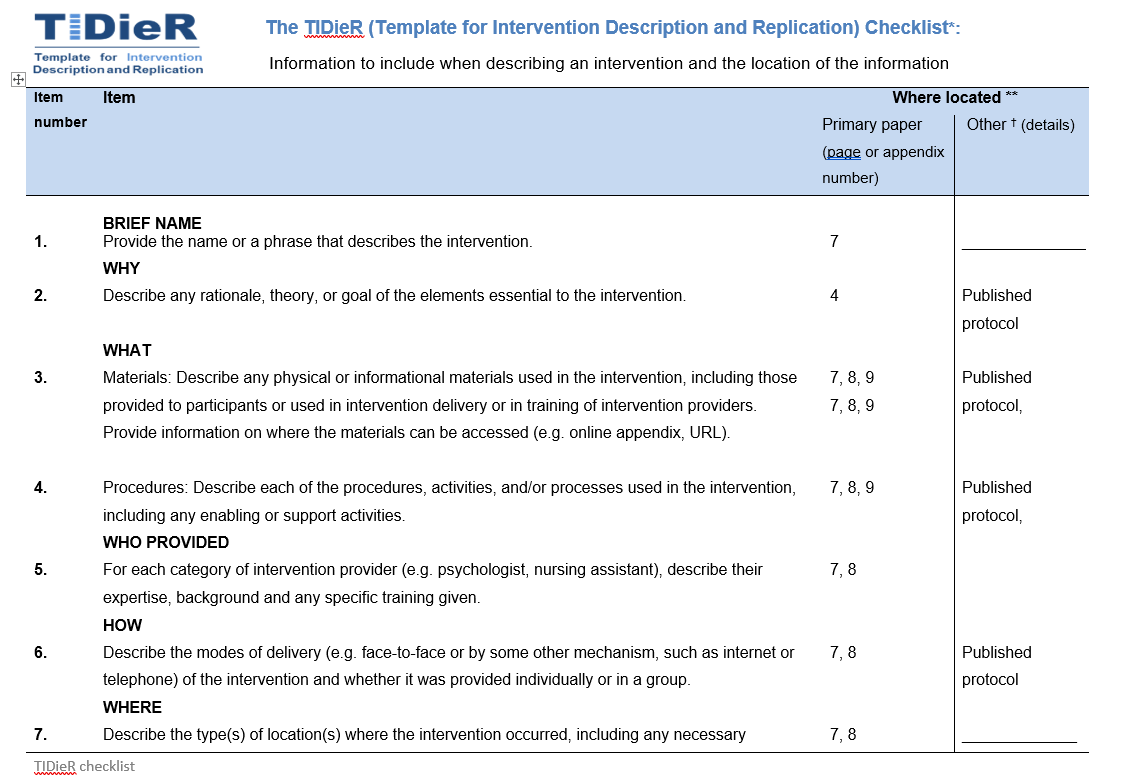


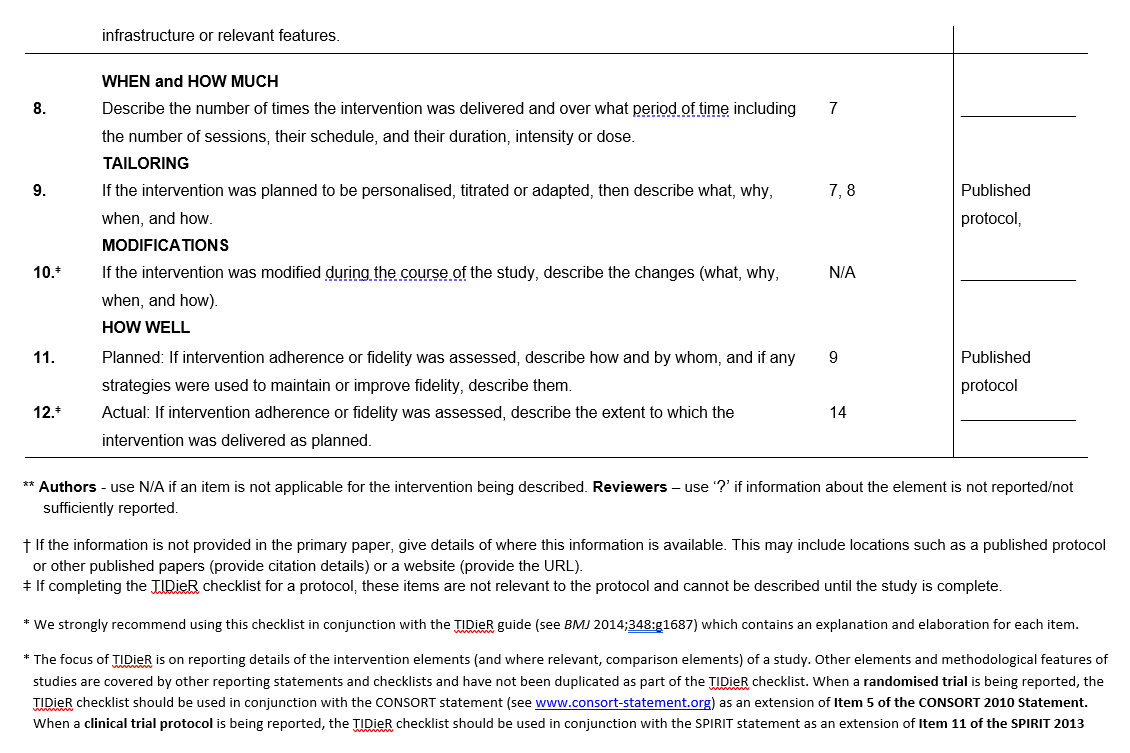

Supplement: Supplemental Material [file YJMT_A_2481605_SM2811.docx]
